# Is Field-sowing of Hybrid True Potato Seeds Feasible for Seed or Ware Potato Production under Dutch Conditions? An Analysis Based on a Review and Twenty Experiments

**DOI:** 10.1007/s11540-025-09889-3

**Published:** 2025-07-17

**Authors:** Luuk C. M. van Dijk, Olivia C. Kacheyo, Wim C. Lieftink, Willemien J. M. Lommen, Paul C. Struik

**Affiliations:** 1 Centre for Crop Systems Analysis, Wageningen University & Research, Bornsesteeg 48, 6708 PE Wageningen, The Netherlands; 2 Solynta, Dreijenlaan 2, 6703 HA Wageningen, The Netherlands; 3 Unifarm, Plant Sciences Group, Wageningen University & Research, Bornsesteeg 48, 6708 PE Wageningen, The Netherlands; 4 Present Address: R&D Plant Based Solutions, Royal Agrifirm Group, Landgoedlaan 20, 7325 AW Apeldoorn, The Netherlands

**Keywords:** Early vigour, Field-sowing, Plantlet establishment, Seed germination, Seedling emergence, Seed system, *Solanum tuberosum* L., True potato seed

## Abstract

The introduction of hybrid true potato seeds (hybrid TPS) offers multiple new ways for seed or ware tuber production in potato, including field-sowing. As opposed to the use of nursery raised seedlings for field-transplanting, which is extensively studied and reported as a practical option for seed or ware production, field-sowing of hybrid TPS is a promising opportunity of which the feasibility for use has not been exhaustively investigated. Field-sowing may be more cost effective as it does not require nursery seedling production. This paper assesses the possibilities of using a field-sown hybrid TPS cultivation system for seed and ware production. First, the critical phases of the system to come to yield formation are defined, namely seed germination, seedling emergence, and plantlet establishment, based on a review. Second, observations and results collected in 20 studies over the course of 4 years indicate that various factors including seed quality and treatment, physical soil properties, soil moisture, weather, and the application and timing of crop management practices all significantly influenced the success of a field-sown crop. The uptake of field-sowing of hybrid TPS for seed or ware tuber production will benefit from improvements in plantlet establishment ratios by improved seed quality and enhanced agronomic practices and technologies. Genetic improvements are beneficial to trigger yield increase in general, and specifically enhance the crop’s early vigour. An important aspect in shaping the future for potato as a field-sown crop is the need to support growers with advice based on experimentation with field-sown TPS in addition to grower’s own experiences growing potato from hybrid TPS or other small-seeded field crops.

**Supplementary Information:**

The online version contains supplementary material available at 10.1007/s11540-025-09889-3.

## Introduction

In potato production, the use of hybrid true potato seeds (hybrid TPS) overcomes the constraints of the conventional seed tuber–based production system (Lindhout and Struik 2023; den Braber et al. 2023). Compared with the open-pollinated (OP) TPS-based system (Wiersema 1984; Almekinders et al. 1996, 2010), the hybrid TPS–based system boasts high uniformity. When using hybrid TPS, the number of field-based multiplications can be significantly reduced (Lindhout et al. 2011; van Dijk et al. 2021) and the total costs and losses during storage and transport will be significantly lower than those of the conventional seed tuber system (Struik and Wiersema 1999, Table 1). Due to the high multiplication rate of TPS, only a few seasons are necessary to attain large quantities of seed tubers in the desired tuber size classes (Kacheyo et al. 2024a). Moreover, most diseases that might be transmitted from generation to generation when using clonal multiplication are not transmitted by TPS.

**Table 1:** Opportunities and challenges for potato multiplication with field-sown hybrid TPS vs. seed tubers

	**Use of true potato seed**	**Use of seed tubers**
Seed health	Hardly any seed-borne diseases transfer via true potato seed	Carrier of tuber-borne diseases resulting in seed tuber degeneration over time
Storage and transport	Small volumes of hybrid TPS	Large volumes of bulky seed tubers
	Dormant, long-term storage in dry stage	Highly perishable and physiologically active
Planting	Seed imbibition initiated by moisture which allows flexible start of crop cycle, when field conditions permit	Start of crop cycle mostly depending on physiological activity of seed tuber and temperature, as initiators for sprouting
	Light-weighted machines, no additional labour for sowing operations needed	Heavy machines, often with additional labour for filling of planting bunker
	Allows for precision seeding	Precision planting possible based on tuber size, not for sprouts per unit area
Initial growth	Slow initial growth due to small seed size with little available nutrients	Vigorous growth due to large meristems and reserves, like sugars, nutrients
	Sensitive to fluctuations of soil moisture and temperature in topsoil due to small initial root system	Self-sufficient in moisture from its mother-tuber during initial growth and rapid take-over by root system
	Novel system needs testing on crop development and potential failure risks	Risks are well established

To produce the first-generation tubers (seedling seed tubers or ware tubers) from hybrid TPS, several cultivation pathways have been suggested including field-transplanting of greenhouse-raised seedlings and field-sowing of TPS (Tuku 1994; Almekinders et al. 2010; van Dijk et al. 2021). The use of field-transplanted seedlings for seed tuber or ware production has recently been proven a feasible alternative to the conventional seed tuber–based system (van Dijk et al. 2021; Kacheyo et al. 2024a). However, it has still not been largely investigated if and how a practical hybrid TPS–based field-sowing system could work, and how feasible that could be for seed tuber or ware potato production (van Dijk et al. 2021, 2022a; Kacheyo et al. 2024a). This study will answer the question whether field-sowing of hybrid TPS can be a feasible alternative for seed tuber or ware production in potato; it will assess the practical feasibility—under normal Dutch conditions—taking into account essential lessons learned from field experiments.

### How Could Field-sowing of True Potato Seed (TPS) Fit in the Potato Seed System?

Similar to the use of field-transplanted seedlings (Kacheyo et al. 2024a), field-sowing of hybrid TPS introduces multiple advantages and the possibility of large-scale potato production at lower costs than with the current clonal system (Table 1). Field-sowing also circumvents the additional costs accrued in transplanting systems (with field-transplanted seedlings) where technological infrastructures for seedling production (often greenhouses), additional labour and seedling tray costs, and transportation are required for transplant production and use. Since seeds are field-sown, the critical aspects in the cultivation pathway of transplants, such as nursery seedling production, field establishment, and transplant shock mitigation, are not relevant. This makes the field-sowing system potentially more cost-efficient than the transplant- and seed tuber–based systems.

**Fig. 1:**
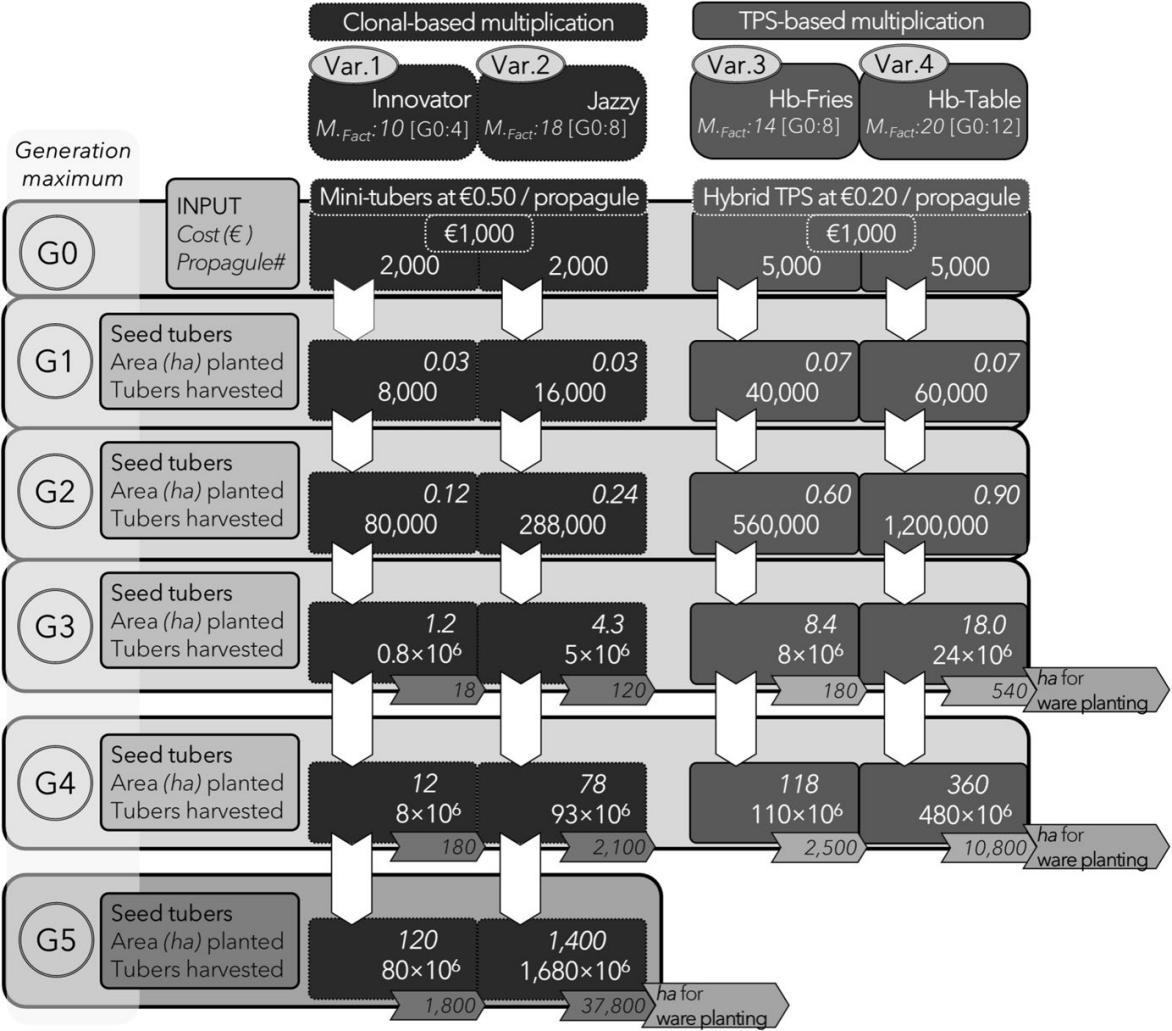
A hypothetical schematic example to compare the **clonal-** and **hybrid TPS–based multiplication scheme**, using four different varieties (***Var1-4***). Each variety has its own multiplication factor (***M.***_***fact***_). As described by Kacheyo (2024), the varieties **Innovator**, **Jazzy**, and **Hb-Table** have multiplication factors of 4, 8, and 12, respectively, when grown from **G0** and 10, 18, and 20, respectively, from **G1** onwards. The variety **Hb-Fries** has a ***M.***_***fact***_ of 8 grown in **G0** and 14 when grown from **G1** onwards (adapted from van Dijk et al. 2021). The following assumptions are included in the scheme: Both systems start with an investment of **G0** starting material of € 1000, whereby the average cost per propagule was set at € 0.50 for mini-tubers and € 0.20 for hybrid TPS, based on the seed costs of hybrid tomato (van den Berg 2024). The ***plant density*** for seed tuber production is set at 66,667 plants/ha. A ***plant density*** of 44,444 plants/ha was used to calculate the covered hectares of ware production derived from the **G3**, **G4**, or **G5** materials of the respective varieties from the seed tuber multiplication. Comparing the French fries type varieties (**Innovator** and **Hb-Fries**) in both systems, the hybrid TPS–based variety can serve with **G4** material already 2500 ha of ware production, compared with 180 ha for the clonal-based variety. For the table type varieties (**Jazzy** and **Hb-Table**), the hybrid TPS–based one can serve 10,800 ha of ware production compared with only 2100 ha for the clonal-based variety in **G4**

Almekinders et al. (1996) elaborated that production costs of 1 ha of seedling tubers via field-sown TPS might be similar to other field-sown vegetables like onion, carrot, and lettuce. According to Almekinders et al. (1996), producing G1 seedling tubers via field-sown TPS could be at lower, similar, or slightly higher cost—depending on yield and multiplication rates—compared with basic seed tubers. Under Dutch conditions, a hybrid TPS–based multiplication scheme might be able to compete with the current clonal system, depending on multiplication rates and cost of the G0 starting material, i.e., hybrid TPS or mini-tubers for the respective systems. Figure 1 gives a hypothetical scheme to get an impression of how field-sown hybrid TPS–based multiplication schemes can quickly generate high numbers of seed tubers—at lower cost per produced tuber—compared with clonal schemes. Based on the hybrid TPS–based system, seed tuber multipliers can economically sell seed tubers for ware production which were in the field for fewer generations, and thus are—theoretically—healthier (cf. Thomas-Sharma et al. 2016). Following Fig. 1, a shorter and more agile potato seed system is theoretically feasible.

### How to Study Field-sowing of TPS in a Practical Setting?

Besides the work with open-pollinated TPS during the 1970s, 1980s, and 1990s (Dayal et al. 1984; Wiersema 1984; Pallais 1987; Almekinders 1995; Almekinders et al. 2010; Buckseth et al. 2022), little research was conducted to test the feasibility of a potato cultivation system based on field-sown (hybrid) TPS (Martin 1983a,b). The innovation of hybrid potato breeding allows to re-open the debate (Jansky et al. 2016) if, and how, field-sowing of (hybrid) TPS is a realistic and practical pathway for seed tuber or ware production. The main aim of the TPS trials discussed in the present study was to explore and lay a foundation for research in cultivation systems for diploid hybrid potato, and to explore the practical feasibility of a field-sown system for potato production from hybrid TPS. This large and diverse set of trials focussing on the hybrid TPS field-sowing system was part of a larger programme, POTAREI (2016–2020). This NWO-funded programme explored scenarios for novel and improved cultivation pathways for diploid hybrid TPS, using inbred lines and experimental hybrid TPS derived from the hybrid breeding programme of Solynta (Stemerding et al. 2023).

In the present paper, key success factors for a field-sown crop will be discussed as was done earlier for field-transplanted hybrid potato seedlings (van Dijk et al. 2021; Kacheyo et al. 2024a). In the section ‘Development of a Field-sown TPS Crop’, the most critical challenges of the system will be identified and discussed via the most critical crop phases for successful yield formation of the TPS-based crops: *seed germination*, *seedling emergence*, and *plantlet establishment*. The section ‘Hands-on Trials to Improve Yield from Field-sown TPS’ shows the major findings and practical experiences attained during the course of the trials, including insights on plantlet establishment successes and attained yields in the trials. Additionally, the most important practices to guarantee success are summarised. Finally, the section ‘Practical Improvements Enhancing a TPS-based Field-sowing System’ provides suggestions for future developments to improve the described hybrid TPS field-sown system by extrapolating practical experiences from field-sown vegetables—like onion, leek, and carrot—and small-seeded arable crops—like sugar beet and chicory—combined with novel agronomical technologies and breeding. The paper ends with a conclusion section.

## Development of a Field-sown TPS Crop

The three major crop phases which field-sown TPS should pass before it successfully can start the yield formation phase of a seed tuber or ware crop are (1) *seed germination*, (2) *seedling emergence*, and (3) *plantlet establishment*. Multiple varying factors influence the success in each of these phases, and, in turn, could lead to the success or failure of the whole field-sown system (Table 2). This section introduces and specifies these three phases by explaining the influencing factors and challenges associated with each phase, as well as possible steps to mitigate the challenges. The section ends with the yield formation and cultivation management options to manipulate yields in a field-sown TPS crop.

**Table 2:** Description of the main factors influencing success or failure in the three critical phases of a field-sown hybrid TPS potato crop before it starts its yield formation phase

Critical phase	Factor of influence	Explanation of the critical aspects
**Seed germination**	Seed vigour, seed-to-seed variation	Individual differences between seeds might inhibit uniform germination
	Available moisture	As TPS are dry organs, sufficient moisture is needed to trigger and maintain germination and growth
	Temperature	Should not exceed development limiting levels
**Seedling emergence**	Sowing-bed preparation	A decent sowing bed is key to fast and uniform seedling emergence and soil moisture maintenance for initial growth. Disturbing soil layers should be avoided
	Sowing depth	Too deep sowing depletes seedlings reserves, too shallow sowing limits available moisture before emergence
	Available moisture	Soil moisture levels should remain within the limits to maintain proper growth
	Temperature	Should remain within critical levels to maintain development
**Plantlet establishment**	Sowing-bed preparation	Proper bed preparation is key to allow seedling root development and maintain sufficient soil moisture
	Available moisture	Sufficient moisture is needed to maintain growth
	Temperature	Should remain within the critical levels to maintain growth
	Available radiation	Is necessary to provide assimilates for plant growth
	Available nutrients	Are necessary to ensure plant growth
	Speed of establishment	Slow establishment makes seedling vulnerable to infections by surrounding pests and diseases, and to weed competition

### Seed Germination

When small-sized TPS is field-sown, the soil moisture—if available—will induce the physiological process of *germination* as the seed is able to imbibe water, ensuing hydration of enzymes and stored reserves like proteins, carbohydrates and fats (Woodstock 1988). As a result, the radicle will emerge from the seed coat (BBCH-005; Kacheyo et al. 2021), developing into roots during the next plant physiological phase, followed by the hypocotyl breaking through the seed coat (BBCH-007; Kacheyo et al. 2021). One of the key factors of success is sufficient availability of moisture during the entire process of *germination*. TPS cannot rely on its own moisture like seed tubers, nor is it able to take up large quantities of moisture during the hydration phase—as reserve until the initial root system can take over—as in large-seeded crops. A well-prepared sowing bed is key to ensure that the seed(ling) is able to maintain its access to the necessary soil moisture (Sanchez 2019). The sowing bed should (1) prevent the received water from easily draining to deeper layers, (2) ensure sufficient capillary rise from deeper soil layers, (3) prevent the sowing bed from easily drying out (Schoneveld 1993), and (4) prevent large temperature fluctuations as much as possible (Villalobos et al. 2016).

### Seedling Emergence

The period from the hypocotyl breaking through the seed coat (BBCH-007) up to the moment that the cotyledons break through the soil surface (BBCH-009) is considered the emergence phase of the hybrid seedling (Kacheyo et al. 2021). Observations on both *germination* and *emergence* in field-sown TPS showed that seedlings started to germinate but, in some cases, could not successfully emerge from the soil, especially when sown deeper in the soil. Deeper soil layers are less prone to drying out; however, sowing deeper prolongs the process of the hypocotyl growing towards the soil surface (BBCH-008), resulting in endosperm depletion before the cotyledon leaves can start to photosynthesise and the seedling can become autotrophic. The structure of the sowing bed is another critical factor influencing the success of *seedling emergence* (Sanchez 2019): a too loose sowing bed frustrates the seedling from reaching its needed moisture whilst a too compact sowing bed obstructs the cotyledons breaking through the soil surface. Lastly, the seed-to-seed variation also plays an important role in the speed of *emergence* (Benjamin 1984; Pallais 1987). Even though TPS is genetically identical, seed size—within a genotype—varies, especially when seed lots are not properly calibrated, cleaned, and graded.

### Plantlet Establishment

**Fig. 2:**
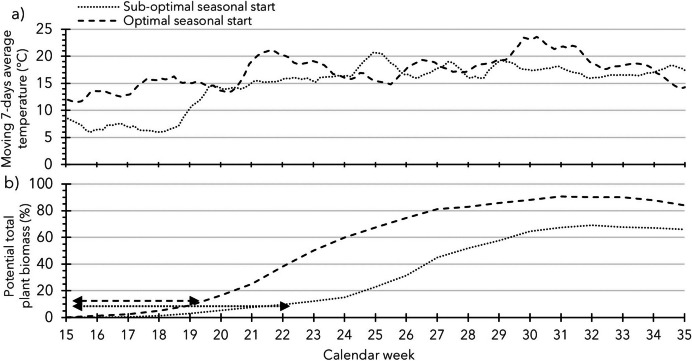
Theoretical example of two contrasting seasonal starts (sub-optimal and optimal) of the cropping cycle (in Calendar Week 15, 2^nd^ week of April) under Dutch conditions. Panel **a** depicts the moving 7-day average temperatures under the two conditions, derived from two theoretical data sets of daily maximum and minimum temperatures (Fig. S1). Panel **b** depicts for both seasons the (hypothetical) biomass accumulation over time for a field-sown hybrid TPS crop. The arrows indicate—more or less—the durations of the *plantlet establishment* phase from the moment of *seedling emergence* up to the growing phase when the total biomass starts to increase more rapidly

*Establishment* of the *emerged seedling* is the next critical phase (Almekinders et al. 2010). *Plantlet establishment* starts from BBCH-100 (onset of leaf development; Kacheyo et al. 2021) up to the commencement of rapid accumulation of above-ground biomass. The newly *emerged seedlings* are vulnerable as they need to acclimatise to above-ground field conditions and switch from dependence on seed reserves to photosynthesis from their first tiny leaves to accommodate leaf appearance, leaf expansion, and stem growth. The field conditions—mild or harsh—during *plantlet establishment* are the major determining factors of the speed of the above-described processes, and thus seedling biomass accumulation, and for the duration of the *plantlet establishment phase*. Figure 2 illustrates two contrasting seasonal starts—temperature-wise—during the *plantlet establishment phase*: (1) the optimal start results in a fast *establishment* and (2) the sub-optimal in a slow *establishment*.

Soil moisture and incoming radiation can also influence *plantlet establishment*. Moreover, the longer *plantlet establishment* takes, the more sensitive seedlings are to pests and diseases, possible yield reduction as well as increased cost for crop protection. Besides, during *plantlet establishment*, weeds emerge and establish themselves in the TPS crop. As weeds are mostly well adapted to harsh field situations, the competition will be strongest in case of slow *plantlet establishment* (Melander and Rasmussen 2001; Melander et al. 2005).

Speed of *plantlet establishment* is also important in relation to crop growth and development (Benjamin 1984; Pallais 1987). Late sowing and delayed crop growth and development do not align with the availability of essential growth resources such as light and soil nutrients.

### Yield Formation and Yield

After plant establishment, biomass accumulation per plant during initial crop growth is slower in a TPS crop compared to a seed tuber–grown plant (Pallais 1987), mainly caused by the single-stemmed habitus of field-sown plants, similar to transplanted hybrid seedlings (Kacheyo et al. 2021; Gu et al. 2024). The success of a field-sown hybrid TPS crop will ultimately be quantified by the yield—fresh tuber yield, or number of tubers in a desired size category—at the end of the crop cycle.

Cultivation management options can be applied to single-stemmed hybrid potato plants to avoid yield reduction compared with multi-stemmed plants from seed tubers (Kacheyo et al. 2024a, b). Examples are the manipulation of plant and (even) row spacings—plant density (van Dijk et al. 2022a; Kacheyo et al. 2024b)—, stepwise earthing up of the ridge (van Dijk et al. 2021), and sowing in beds or on a flat field, instead of on ridges (Kacheyo et al. 2024b). Like in many other crops, crop management adjustments by the grower are key to steer yield and quality towards a desired potato production outlet. Concurrently, timing of sowing (van Dijk et al. 2022b) is an important crop management aspect, as it allows the grower to manipulate the crop cycle length, which is slightly longer for a field-sown crop than for a tuber-grown crop (Almekinders et al. 2010). Both at the start—timing of sowing—and at the end of the season—timing of harvest—the grower has to deal with the local weather conditions which might delay sowing activities as well as advance or delay harvest activities. Especially, increased probability of local extreme precipitation events is foreseen for the Dutch climate (Bessembinder et al. 2023), which might hinder the grower’s field management activities. Due to climate change, early-maturing varieties are preferred (van Dijk et al. 2022b) since crops should be harvested before winter rains set in to allow sowing of a winter catch crop, a legal obligation for large regions in the Netherlands (Adema 2023), which prevents N-leaching.

Multiple field trials involving the manipulation of crop management factors were carried out from 2016 to 2019. In total, 20 (17 field, 3 climate controlled) trials were conducted to unravel the challenges and critical success factors for a novel potato cultivation pathway based on field-sowing of hybrid TPS (see below in the next section). In the field trials, yield varied from 0 to 50 Mg/ha (Table 3). In general, over time, yield in the trials improved, mainly caused by improved crop management due to increased experience; the increased experience of the researchers and field assistance contributed significantly to the success over time. Crop management improvements in the early phases of the cultivation cycle—*germination* and *emergence*—led to the greatest successes.

**Table 3:** Overview of trials on sowing hybrid TPS conducted in the Netherlands from 2016 to 2019. The first column indicates year and trial code (*yy*.*n*.*n*) and locations (***Wag***, ***HBK***, ***ODG***, and ***EST*** = **Wag**eningen University & Research, **H**ilvaren**b**ee**k**,** O**u**d G**astel, and **Est**, respectively). A trial’s overall success—based on learning experiences and/or yield—is ranked as very unsuccessful (–), not successful (-), neutral (±), successful (+), and highly successful (+ +)

Year.trial.code; location	Trial aim	Trial highlights and/or summary (trial was conducted at sandy soil when soil type is not specified)	Estimated % plant survival	Yield range	Trial success
16.1.1; Wag	*First test*	First pelleted TPS; first field-sown established plants	5–10	< 1 kg/plant	+
17.1.1; Wag	*Explorative*	Smaller pellets; first field-sown crop; first successful yield	25–60	15–40 Mg/ha	+ +
17.3.1; Wag. *Climate room*	*Explorative*	Sowing depth and soil moisture are critical factors to steer the success of seedling emergence	7–28	Not measured	+ +
18.1.1; Wag	*Explorative* — timing; row-distance; density	High losses of emerged plants due to herbicide application in first timing treatment	Not observed	20–50 Mg/ha	±
18.1.2; Wag	Sowing depth	Sowing > 3.5 cm is too deep. Germinated TPS ≠ emergence	< 5–20	7–23 Mg/ha	+
18.1.3; Wag	*Explorative* — plant development	*Manual* *sowing*; naked, non-pelleted seeds do not germinate nor emerge, pelleted seeds do both	50–60	Not measured	+
18.1.4; Wag	Sowing depth	*Manual* *sowing*; soil compression negatively influences emergence	Not observed	Not measured	-
18.1.5; ODG	*Explorative* — timing	*Clay soil*; growers’ field trial; emergence < 1%	0	None	–
18.1.6; ODG	*Explorative* — timing	Growers’ field trial; emergence < 1%	0	None	–
18.1.7; HBK	*Explorative* — timing	Growers’ field trial; emergence 50%; plant success ratios varied over time from 0.0 to 0.3; first yields with large tuber sizes (Fig. S4)	0–30	4–14 Mg/ha	±
18.1.8; HBK	*Explorative* — genotypes	Growers’ field trial; emergence < 1%	10–30	No harvest	–
18.1.9; EST	*Explorative* — genotypes	*Clay soil*; growers’ field trial; emergence < 1%	0	None	–
18.1.10; ODG	*Explorative* — genotypes	*Clay soil*; growers’ field trial; emergence < 1%	0	None	–
19.3.2; Wag. *Greenhouse*	Sowing depth	Increased sowing depth decreased emergence; sowing depth of ~ 1 cm most successful	Not observed	Not measured	+ +
19.1.1; Wag	Timing	Environmental conditions around initial seedling phase have clear effect on emergence and plant establishment	8–55	10–40 Mg/ha	+ +
19.1.2; Wag	Sowing density	Increased plant numbers result in increased yields	< 5–60	10–55 Mg/ha	+ +
19.1.3; Wag	Sowing depth	Survival of shallow sown TPS (0–1 cm) depends on soil moisture	< 5–35	3–28 Mg/ha	+
19.1.4; Wag	Sowing system	Increasing plant density on ridges by double rows increases yields	10–35	17–24 Mg/ha	+ +
19.2.1; Wag	Plant development	Phenological effects of propagule types and timing of sowing	50–60	10–20 Mg/ha	+ +
19.3.1; Wag. *Greenhouse*	Plant development	Detailed phenological effects of propagule types on growth and development	Not observed	Not measured	+ +

The higher yields obtained in the field-sown crops were comparable to those obtained from field-transplanted experimental hybrids, as reported by van Dijk et al. (2021; 2022a, b) and Kacheyo et al. (2024b). In the next section, the most important experiences, along with experimental yields, will be shared.

## Hands-on Trials to Improve Yield from Field-sown TPS

In 2016 and 2017, the main aim of the trials (Table 3) was to verify the feasibility of field-sowing using inbred lines and scarcely available experimental hybrid TPS in the respective years. From 2018 onwards, experimental hybrid TPS was used in the trials to examine the influences of important crop management factors such as sowing depth, timing, and density in relation to crop performance and environmental conditions. Success was quantified by the number of *plants established* and yield (Table 3).

The climate-controlled trials (climate room, greenhouse) and most field trials were conducted at the Wageningen University & Research experimental facility Unifarm. Field trials were primarily conducted on sandy soils at Nergena (51° 59′ 40ʺN 5° 39′ 24ʺE). In 2018, six additional field trials were conducted at growers’ fields (Table 3): on clay and a sandy soil in Oud Gastel (ODG, 51° 36′ 03ʺN 4° 29′ 14ʺE), on a light sandy soil in Hilvarenbeek (HBK, 51° 29′ 34ʺN 5° 04′ 45ʺE), and on a heavy clay soil in Est (EST, 51° 50′ 59ʺN 5° 19′ 55ʺE).

**Fig. 3:**
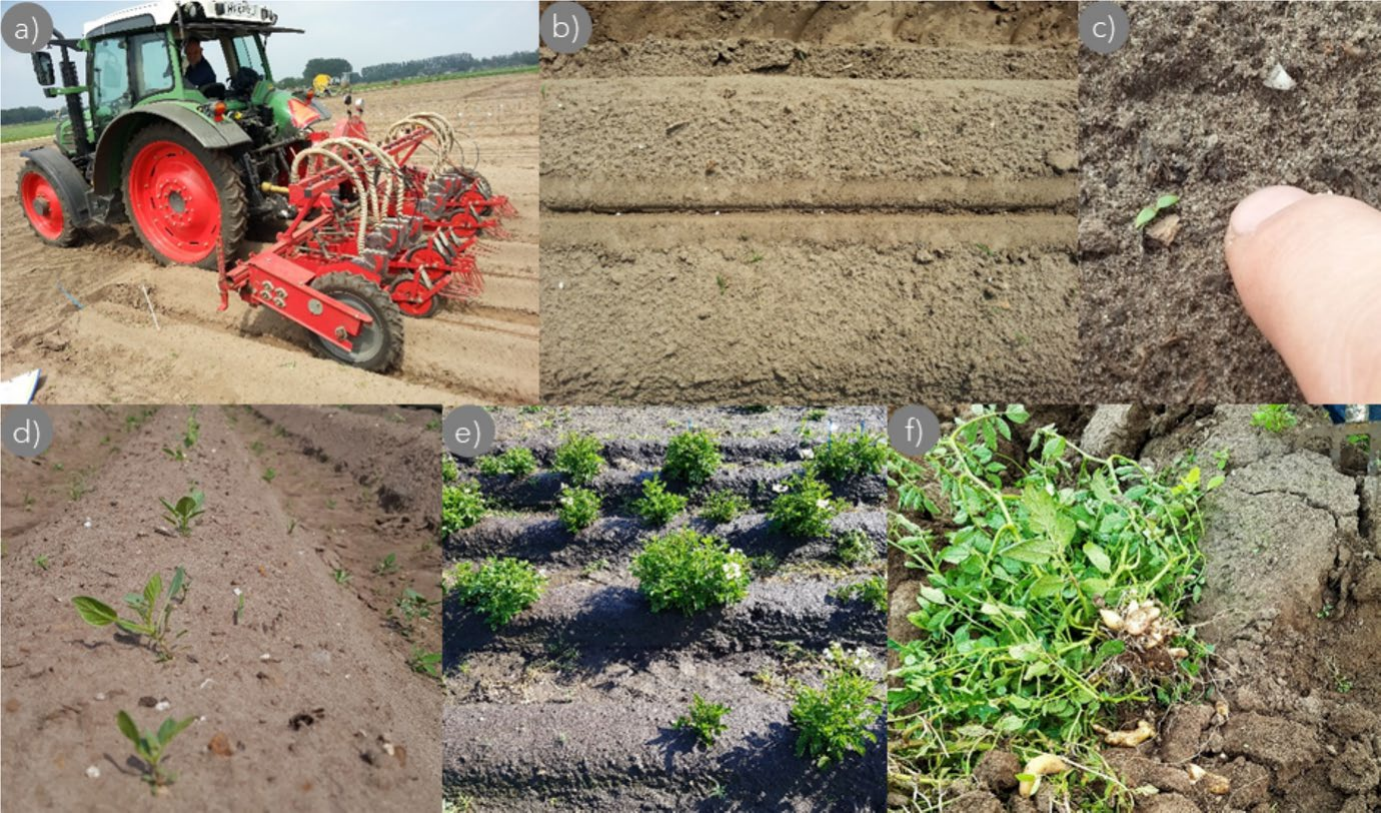
First attempt of mechanically field-sown TPS derived from inbred lines during the 2016 growing season in Wageningen (16.1.1; Table 3). Pelleted seeds (diameter 3–4 mm) were sown with an Accord miniair S pneumatic precision sowing machine. Panels show different moments throughout the season; pictures were taken at *sowing* on 2 June (**a** + **b**), at *seedling emergence* on 11 June (**c**), at *plantlet establishment* on 20 July (**d**), at *yield formation* on 12 September (**e**), and *at harvest* on 14 October 2016 (**f**)

In the trials, sowing-bed preparation was done following standard agricultural practices for small-seeded crops (like sugar beet, chicory, onion, leek, and carrot) for the respective soil types (CTGB 2018). All field trials were sown by a pneumatic Accord miniair S precision sowing machine (Fig. 3a), which is conventionally used for sowing various small-sized, pelleted vegetable seeds (Schoneveld 1993). A standard sowing depth (approximately 1–2 cm) was used for all but the sowing depth trials and was implemented in a sufficiently moist sowing bed. The precision sowing machine placed seeds on a reconsolidated seed horizon to facilitate the capillary rise of water, whereafter seeds were covered with soil and slightly pressed into place (similar to sowing practices for sugar beet, chicory, onion, leek, and carrot). In the absence of rain, additional irrigation—using an overhead sprinkler boom or rotating pivot—was applied to keep the sowing bed moist, similar to the commercial practice for small-sized field-sown vegetables. Below, the most important gained hands-on experiences derived from the conducted trials are shown and discussed.

### Naked vs. Pelleted TPS

Past experiences of Lindhout and colleagues proved that sowing ‘naked’ TPS in the field, without any type of pellet or coating did not work, resulting consistently in 0% *emergence* (P. Lindhout, personal communication, November 2015). To address this challenge, pelleted TPS was used in all field-sown trials from 2016 onwards. In the current study, one trial (18.1.3) focusing on plant development over time included naked seeds, as well as pelleted seeds. Naked seeds did not *emerge*, despite frequent irrigation, but pelleted seeds did. Seed pelleting is the process of adding inert materials to seeds increasing their weight, size, and shape. The *germination* success of the pelleted seeds can be attributed to its material, dried clay (only). The pellet can be seen as an extension of the seed itself, increasing the water-holding capacity of the seed, which benefits the *germination* and *emergence phases*, especially when the surrounding soil contains little moisture (Box 1). It is important to state that abundant (soil) moisture is needed to first moisturise the pelleted seeds to induce the *germination* process. An additional advantage of pelleted seeds is that existing precision sowing machines can be used, which enables a more precise sowing in terms of seed-to-seed distance as well as sowing depth. Hence, pelleting TPS for field-sowing exercises is the first step to ease the processes of sowing, *germination*, and *emergence*, which are critical for *plantlet* *establishment*, crop growth, and yield.

### Soil Moisture and Irrigation

Experiences from raising hybrid potato transplants in a greenhouse nursery, but also from sowing small-seeded open field vegetable crops, show that sufficient available soil moisture in the initial crop phase is important for successful *plantlet* *establishment*. In 2016 and 2017 (Trials 16.1.1 and 17.1.1), irrigation was applied by small rotating sprinklers, fixed on static irrigation tubes, which could be switched on at every moment when needed, followed by a large irrigation boom in the consecutive years.

**Fig. 4:**
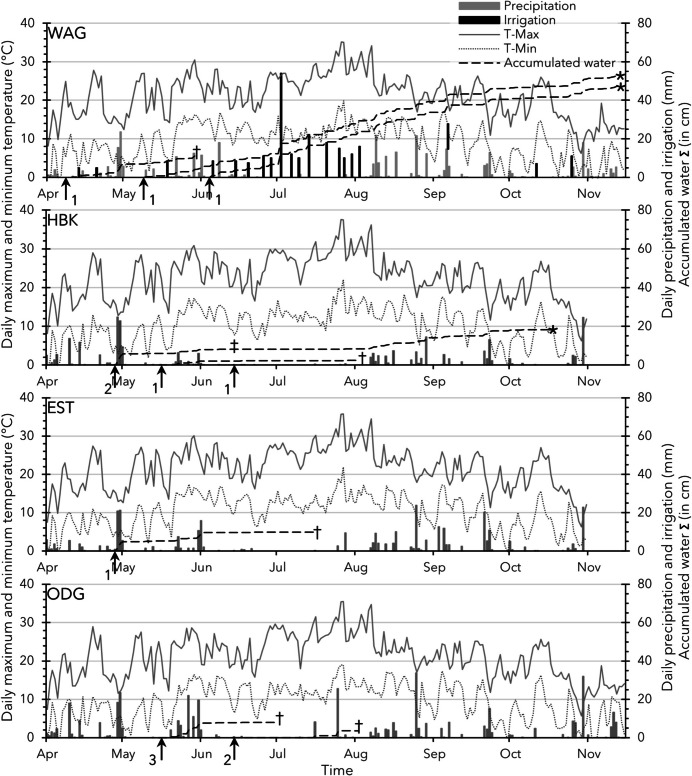
Weather patterns during the 2018 season for the four sowing locations Wageningen (**WAG**), Hilvarenbeek (**HBK**), Oud Gastel (**ODG**), and Est (**EST**), Table 3 Trials 18.1.1 and 18.1.5 until and including 18.1.10. Sowing dates are indicated with arrows, including the number of trials sown at that moment. Compared with normal climatological averages, early spring (February, March) came with much more precipitation. Also in April, the trial field locations received twice as much (almost 100 mm) precipitation as the climatological average of 40 mm. From May onwards—when trials were sown—precipitation was very little. The dagger symbol indicates the observation moment when the few *emerged seedlings* were dead, and the asterisk symbol indicates the harvest moment at full maturity of the crop

The importance of sufficient—and constant—availability of soil moisture in the early crop phases became clear in 2018, when field-sowing was carried out in a researcher-managed trial in WAG (sandy soil, three sowing moments) and three growers’ fields, HBK (sandy soil, three sowing moments, and one demo trial), ODG (clay and sandy soil with—at both soil types—three sowing moments and one demo trial on clay), and EST (clay soil, one demo trial), see Table 3 Trials 18.1.1 and 18.1.5 until and including 18.1.10. After sowing, all locations received very little precipitation compared to a normal spring, as is shown in Fig. 4 together with the applied irrigation. Location WAG received irrigation almost twice a week. For HBK, when necessary, irrigation was applied every 10–15 days for the whole growing season, whilst at ODG and EST it was applied every 15–20 days during the first 2 months after sowing. As a result, from the nine different sowing moments at the three growers’ field locations, only at the HBK field > 10% *plant establishment* was observed—for all sowing dates—and only the crop sown on the first sowing date (showing 50% *emergence* at 20 days after sowing) was harvested. At the locations ODG and EST, *emergence* was < 1%. At WAG, the emergence was not observed in detail due to weed and herbicide-damage challenges; however, a fair share of at least 30% of the sown TPS did emerge.

**Box 1** Size matters: TPS and its pellets.

**Table:** 

From past research, it is known that OP-TPS is significantly smaller and lighter (1–2 mm and 0.75 g per 1000 seeds; Almekinders and Wiersema 1991) compared with the average seed tuber (35 mm, 55,000 g per 1000 seed tubers). The experimental hybrid TPS used in the current trials was even smaller compared with OP-TPS: 0.8–1.5 mm in size and an average weight of 0.37 g per 1000 seeds. In 2016, when conducting the first field-sown trials, pelleted TPS measured 3–4 mm in size, whereby the pellet was about 100 times heavier than the TPS inside. During sowing, it was observed that many ‘theoretical seed positions’ in the ridge contained a seed with a crushed pellet or were empty (so-called missed seeds). The difference between the size of the seed and the intended pellet size made that a lot of pellet-product was needed to reach the final pelleted seed size, resulting in pellets which were often oval or oddly formed with an irregularly shaped surface. For successful precision seeding, almost perfectly round seeds are needed to hold well on the holes (by creating a vacuum from the other side of the sowing disc) in the seeding discs. When seeds are odd-shaped, they either fall too early from the sowing disc—resulting in ‘missing seeds’, which means that there is no seed sown on a particular aimed position in the row—or will be sucked through the holes of the disc, resulting in crushed pellets. Therefore, in the experimental years from 2017 onwards, pellet sizes of the seeds were reduced significantly to 1.8–2.5 mm, whereby the pellet was only 25 times heavier than the seed inside. As a result, pellets did not crush on the sowing discs and the number of ‘missed seeds’ was reduced to a minimum.	

Combining the information about precipitation and irrigation with the sowing dates (Fig. 4) suggests that failure of *emergence* in the growers’ fields was most likely due to a lack of sufficient soil moisture during the initial phases of *seed germination*, *seedling emergence*, and *plantlet establishment*.

### Sowing Depth

Sowing into deeper soil layers is a management practice to ensure prolonged periods of exposure of seed and *emerging seedling* to sufficient soil moisture (Schoneveld 1993). Evaporation leads to a significant decrease in soil moisture in the topsoil layers, with a gradual increase in moisture towards the lower soil layers. Deeper sowing is used for other field-sown crops like maize and beans. Besides an improved chance of fulfilling the seed(ling) moisture requirements, an increased sowing depth also protects young seedlings against cold/frost or bird damage. However, as opposed to large-seeded crops, TPS faces a critical disadvantage when sown into deeper soil layers.

Deep sowing (> 3 cm) of TPS comes with the trade-off of failure of *seedling emergence*. Sowing deeper did not hinder *germination* but led to failure of elongation of the hypocotyl such that seedlings failed to break through the soil surface and died without reaching *emergence stage* (BBCH-100; Kacheyo et al. 2021).

Experiences from sowing depth trials (17.3.3; 18.1.2; 18.1.4; 19.3.2, and 19.1.3) showed that sowing at shallow depths (< 1 cm) often resulted in the success of the early phases of the field-sowing system—*seedling germination* and (sometimes even) *emerging*—but quickly thereafter seedlings died because of a shortage of available soil moisture. As the topsoil layer, especially of sandy soils, is prone to prompt drying out—even when frequently irrigated—*plantlet establishment* at shallow sowing depths mostly failed due to a poorly developed root system in these initial plant stages.

From two trials (17.3.3, 19.3.2) in a controlled environment, it was learned that shallow sowing (0–0.5 cm) resulted in the highest proportion of *plantlet establishment*, compared with various depths up to 4 cm, but only with a constant availability of adequate soil moisture. In these controlled environments, plantlets were not exposed to any wind, and drying out of the topsoil layer could be better avoided than in a real field situation. Combining all insights on sowing depth in a Dutch sandy soil, between 1 and 2 cm deep was seen as the most conducive depth, similar to carrots (Schoneveld 1993). However, proper sowing depth alone cannot guarantee success in a field-sowing system; rather, its interaction with soil type, available soil moisture, and other environmental and biotic factors will contribute to the success.

### Timing of Sowing

Shifting the timing of sowing allows a grower to anticipate field and weather conditions, as well as change the length of the growing season. Due to the slow initial growth of the seedling, early sowing is preferred to advance the cropping cycle; however—under Dutch conditions—an early start is coupled with an increased risk of unfavourable conditions such as frost, or cold weather (van Dijk et al. 2022b) or wetter periods which limit growth and expose seedlings to disease.

**Fig. 5:**
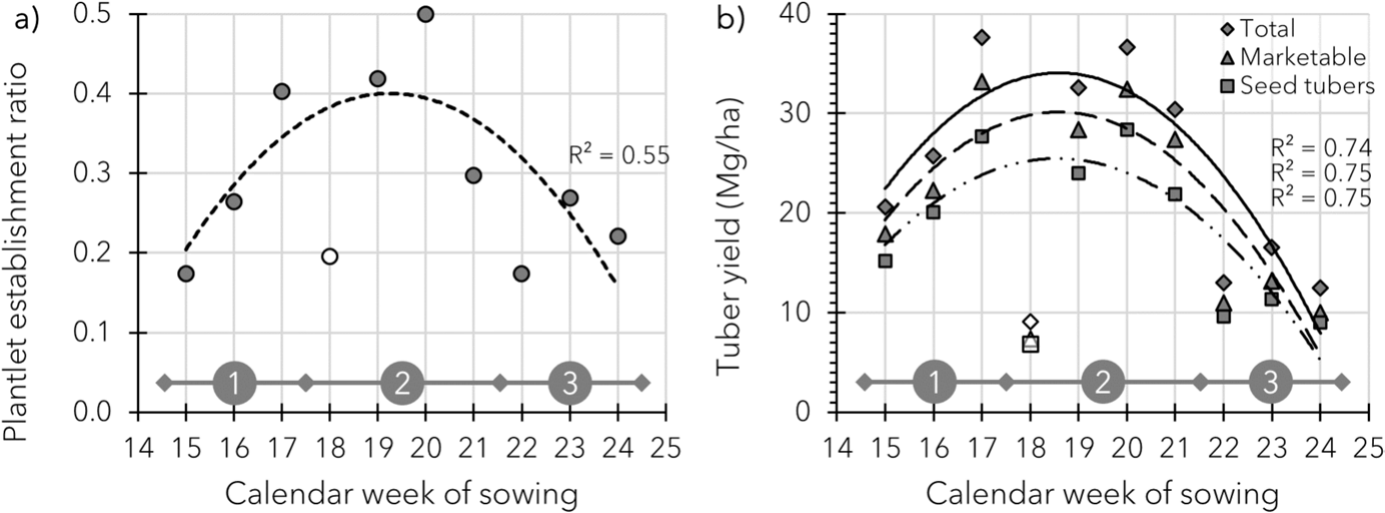
Data of the sowing timing trial of 2019 (19.1.1, Table 3) which involved weekly sowing from Calendar Week 15 until 24 (sowing density: 18.2 seeds/m^2^). *Plantlet establishment* ratio is shown in Panel **a** and produced total, marketable (> 28 mm), and seed tuber (> 28 ≤ 50 mm) yields (harvested on October 7; Week 41) are shown in Panel **b**. In both panels, trendlines (2^nd^-order polynomial) are inserted, including the corresponding *R*^2^-values, based on all data except the outliers for Week 18 (caused by a temperature drop and seedling removal by weeding). The *R*^2^-values for the data sets including Week 18 were 0.35, 0.31, 0.33, and 0.33 for *plantlet establishment* ratio, total, marketable, and seed tuber yields respectively

In 2019, a trial (19.1.1, Table 3) was laid out with 10 weekly sowing intervals from April (Calendar Week 15) until June (Week 24). Irrigation was carried out frequently to avoid water-limiting conditions. The sowing density was fixed at 18.2 seeds/m^2^ by a 0.50 × 0.11 m *row* × *seeding* distance. The *plantlet establishment* ratio (plantlets established/seeds sown) ranged between 0.17 and 0.50, as shown in Fig. 5a. Over the first few sowing weeks, there was a clear increase in *plantlet* *establishment*, and in the final weeks of sowing, the *plantlet* *establishment* showed a decrease (indicated in Fig. 5 with Periods 1 and 3 respectively). When sown in Weeks 17 to 21 (end of April to end of May, Period 2 in Fig. 5), the highest numbers of sown seeds were able to *establish* *plantlets*, except when sown in Week 18. The low number of *established* *plantlets* from Week 18 can be explained by a combination of a drop in temperature just after *emergence* and a crop management failure (weeding, which caused undesired seedling removal during the *plantlet establishment phase*), which can be seen as a challenge in crop management of field-sown TPS.

At the end of the season, on 7 October, the whole trial was harvested. Total tuber fresh weight was on average 23.5 Mg/ha, ranging between 9.1 and 37.7 Mg/ha. Following the produced yield per sowing moment in Fig. 5b, roughly three phases in time could be identified: (1) in the first 3 weeks of sowing, at every consecutive week the final tuber yield increased from 20.6 to 37.7 Mg/ha, followed by (2) a more or less stable yield of 34 Mg/ha from Sowing Weeks 17 to 21, whereafter (3) the yield dropped from Sowing Week 22 onwards to 14 Mg/ha. Sowing Week 18, as previously discussed for *plantlet establishment*, did not follow this trend. In terms of marketable yield (> 28 mm), 17.7–33.2 Mg/ha was measured in Phase 1, followed by 30.4 Mg/ha when yield stabilised (Phase 2), whereafter marketable yield dropped towards 11.4 Mg/ha in Phase 3 (Fig. 5b).

When combining the produced yield with the number of *established plants*, *plantlet establishment* can be considered as a major factor for good yields with field-sown TPS. However, in the last phase, from Week 22 onwards, not just the number of *established* *plants* became lower, but also the duration of the growing period was decreased to a level that the crop could not keep up with the yields of the earlier sown crops. From this example, sowing between mid-April and end of May will result in the highest final yields.

### Sowing Density

**Fig. 6:**
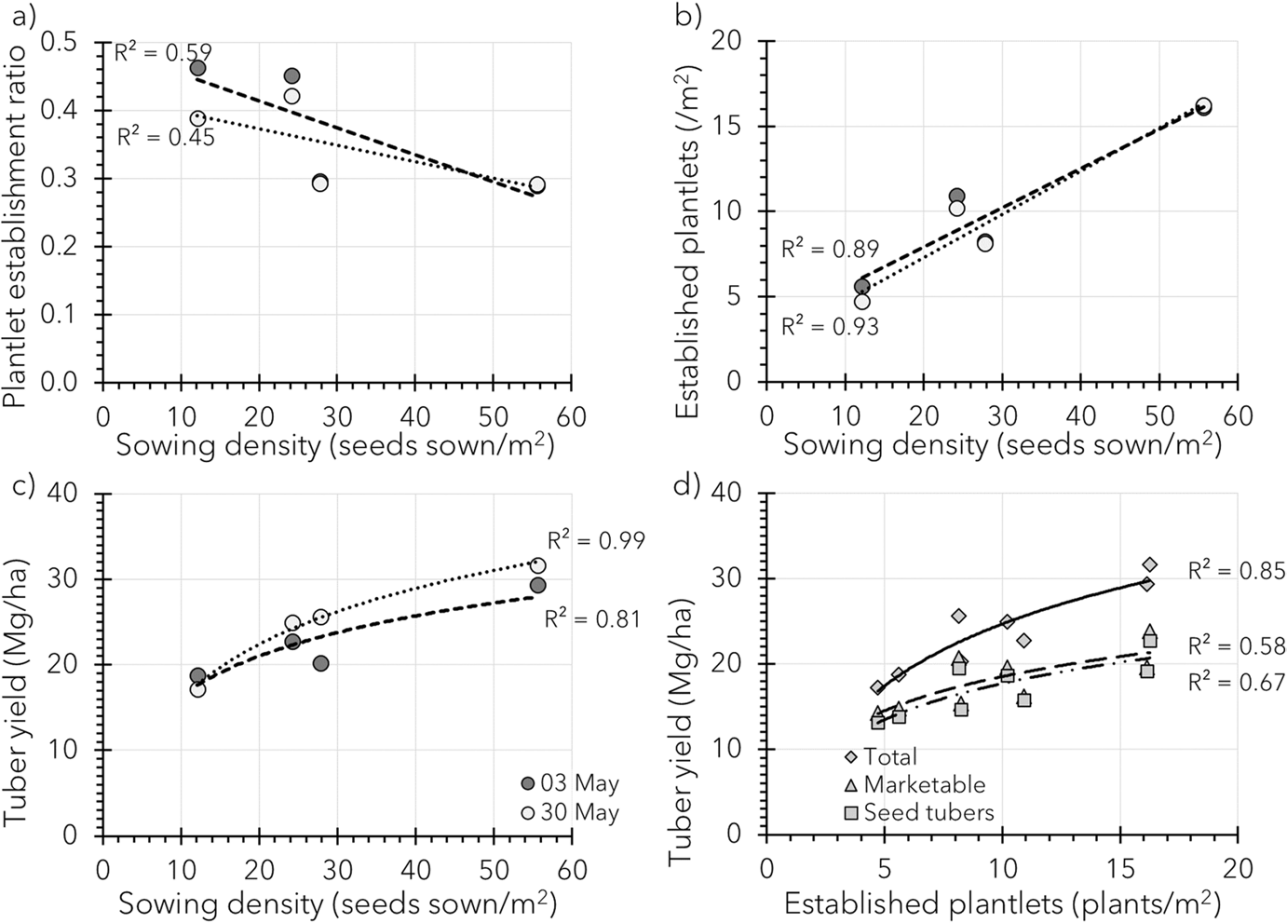
Data of the first explorative sowing density trial, sown in 2017 (17.1.1, Table 3). The trial was repeated in time, sown on 3 and 30 May and sown at four different densities of 12.1, 27.8, 24.2, and 55.6 seeds/m^2^. For all panels, trendlines (linear for Panels **a** and **b** and logarithmic for Panels **c** and **d**) are inserted, including the corresponding *R*^2^-values to give an idea about the observed relationships. Plantlet establishment ratios plotted over sown densities for both sowing dates are shown in Panel **a**. The number of *established plants* against the number of sown seeds per unit area is shown for both dates in Panel **b**. The total yield on both sowing dates against sowing density is shown in Panel **c**. The produced total, marketable (> 28 mm), and seed tuber (> 28 ≤ 50 mm) yields against the number of established plantlets, without discriminating between sowing dates, are shown in Panel **d**

From the first trials conducted in 2017 and 2018, the number of *established plantlets* was found to be one of the major yield-determining factors. The larger the number of *established plantlets* (per unit area), the higher the tuber yields in general. In 2017, these insights were derived from an explorative trial with experimental hybrid genotypes, which studied the effects of manipulation of row and seeding distance (sowing density); see Fig. 6. Row distances were chosen such that mechanical earthing up of the plantlets and mechanical weeding were still possible; see Box 2.

The trial in 2017 was sown on 3 May and on 30 May, to have a replicate in time and to spread potential risks of unfavourable weather conditions in the sensitive period from *germination* to *plantlet establishment*. It was aimed to achieve sowing densities of 12.5, 25, and 50 seeds/m^2^, equal to transplant densities used by van Dijk et al. (2022a). In practice, with row spacings of 75 and 37.5 cm, four different sowing densities of 12.1, 27.8, 24.2, and 55.6 seeds/m^2^ were applied using the row × seeding distances 0.750 × 0.110 m, 0.750 × 0.048 m, 0.375 × 0.110 m, and 0.375 × 0.048 m (Fig. 6).

The trial clearly showed that the linear relationship between number of TPS sown (per m^2^) and *established* *plantlets* (per m^2^) was relatively strong with *R*^2^-values of 0.89 and 0.93 for the sowing on 3 and 30 May, respectively (Fig. 6b). Total produced tuber yields varied between 17.2 and 31.6 Mg/ha (Fig. 6c, d), whereas an increased sowing density resulted in a higher yield (*R*^2^-values of 0.81 and 0.99 for the sowing dates of 3 and 30 May, respectively).

**Fig. 7:**
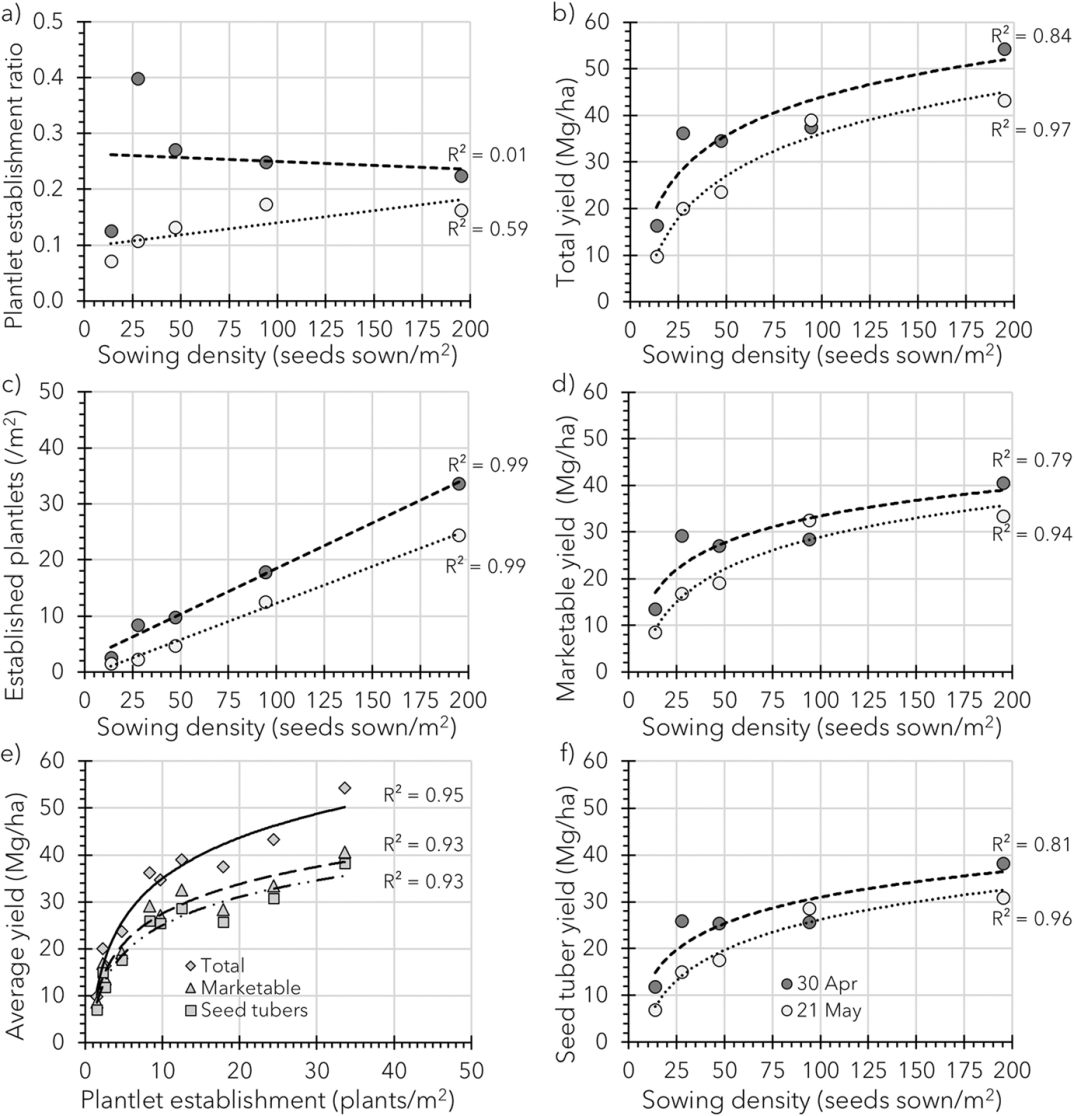
Data of a sowing density trial (19.1.2, Table 3) conducted in 2019 which was repeated twice in time, sown on 30 April and 21 May. It was aimed to achieve sowing densities of 12.5, 25, 50, 100, and 200 seeds/m^2^, equal to transplant densities used by van Dijk et al. (2022a). In practice, with row spacings of 50, 25, and 12.5 cm, five different densities of 13.8, 27.6, 47.1, 94.1, and 195.1 seeds/m^2^ were applied using the row × seeding distances 0.500 × 0.145 m, 0.250 × 0.145 m, 0.250 × 0.085 m, 0.125 × 0.085 m, and 0.125 × 0.041 m, respectively. For all panels, trendlines (linear for Panels **a** and **c** and logarithmic for Panels **b** and **d**–**f**) are inserted, including the corresponding *R*^2^-values to give an idea about the observed relationships. Panel **a** shows for both sowing dates the plantlet establishment ratios (plant nr established/seed nr sown) plotted against sowing densities. A strong relation between sowing density and *established plantlets* per unit area is shown for both sowing dates in Panel **c**. The produced total, marketable (> 28 mm), and seed tuber (> 28; ≤ 50 mm) yields averaged over both dates are shown in Panel **e**, followed by the produced total, marketable, and seed tuber yield per sowing date in Panels **b**, **d**, and **f** respectively

Based on these 2017 results, a sowing and seedling density trial was conducted in 2019, with the assumption that under similar sowing, *germination*, and *emergence* conditions, higher sowing densities would result in more *established plantlets* (per m^2^). The trial contained five different densities and was sown on two different dates for risk-spreading reasons (Fig. 7).

The observed *plantlet establishment* ratios were in general low (0.253 and 0.129 for sowing dates 30 April and 21 May, respectively), and did not show a clear trend, especially not in the trial sown on 30 April (*R*^2^-values 0.01, Fig. 7a). Thus, the number of *plantlets established* (per m^2^) as a result of an increased sowing density clearly increased (*R*^2^ = 0.99) for both sowing dates (Fig. 7c). Likewise, clear (logarithmic) trends were observed for tubers yields, which markedly increased over an increased sowing density for total, marketable (> 28 mm), and seed tuber (> 28 ≤ 50 mm) yields (Fig. 7b, d–f).

**Box 2** Weed management and hilling: how to deal with it in field-sown trials and future crops?

**Table:** 

**Fig. 8:**
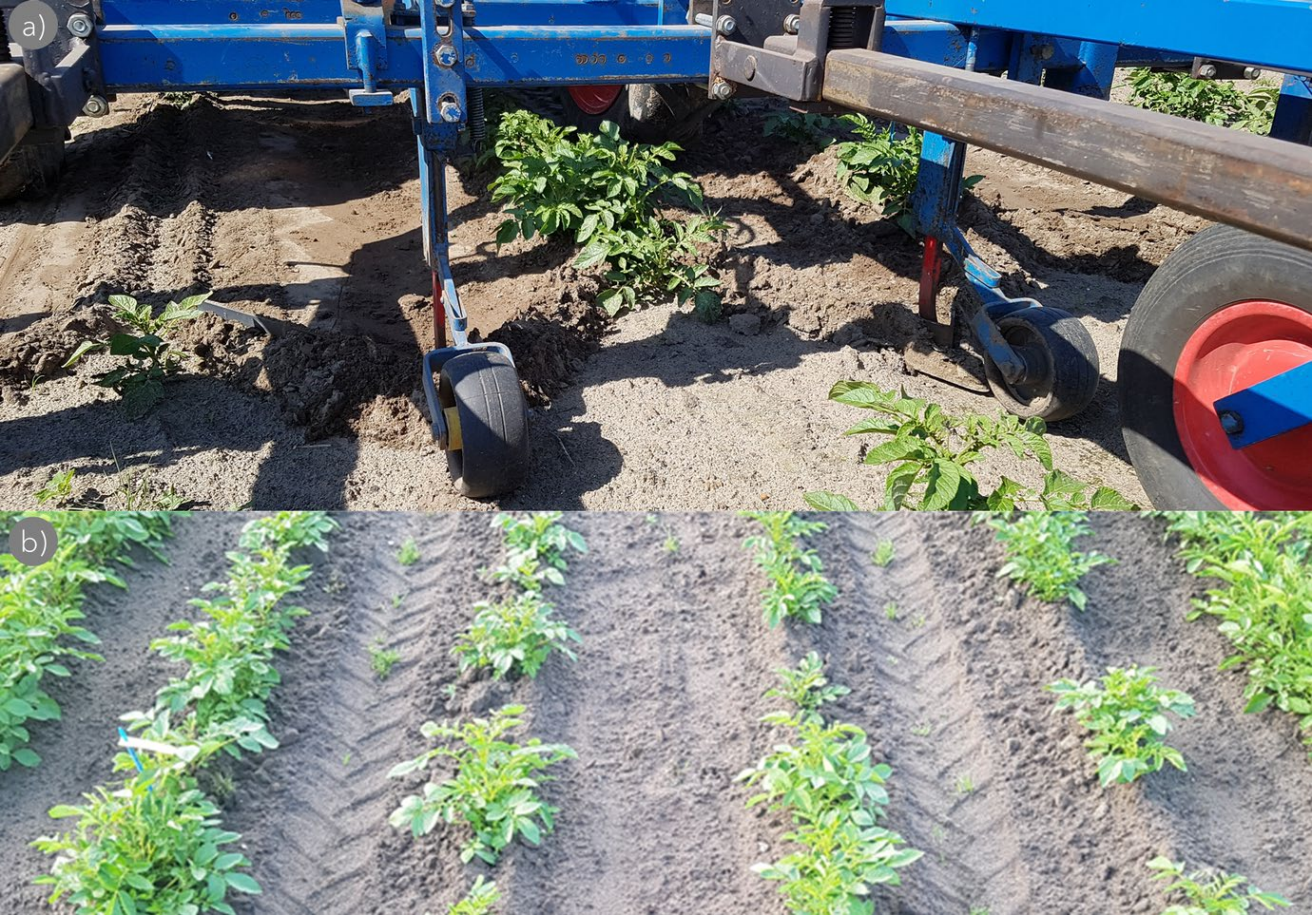
Mechanical weeding (**a**) by using modified blades and the general outlook (**b**) of field-sown rows (at 75-cm row distance) with some earthen-up soil from a mechanical weeding in 2017

Weed control is a critical aspect in a field-sown crop (Cloutier et al. 2007; Ascard and Fogelberg 2008), especially during the phases of seedling emergence and plantlet establishment due to slow growth rates of the crop. Slow initial growth makes the seedlings less competitive to the weeds; consequently, weeds are more likely to overgrow the seedlings and cover the soil much faster. Applying herbicides to control weeds is common practice in potato cultivation. However, as the TPS used in these early trials were experimental hybrids, they were not yet tested for herbicide tolerance. For example, in 2019, it was observed that the experimental hybrid seedlings used were very sensitive to an application of Basagran, which resulted in an almost complete crop loss. Therefore, herbicide applications were reduced to a minimum. Nevertheless, the limited use of herbicides in the trials did not mean that hand-weeding was the only option. In the trials on flat beds, weeding was done between the rows using a modified hoe-weeder which allowed to weed as close as 2.5 cm next to the plant in an early seedling stage. In later crop stages, this was adjusted to 5 cm to prevent cutting of the plant’s stolons. Because individual field trials were of small size, the non-mechanical-weeded area close to the plants was cleaned by hand. However, so-called finger-weeders or brush-weeders—as often used in vegetable crops—can also do the job (Melander et al. 2005; Cloutier et al. 2007), especially when hand-weeding is too costly. A positive side-effect of carrying out mechanical weeding with modified hoe blades was the additional soil brought to the field-sown plants, resulting in small hills, supporting the basal stems of the plants, as shown in Fig.8. Depending on machine adjustments, speed, and the number of weedings, the hills reached a final size between 5 and 10 cm at the moment of full canopy cover. In 2019, one trial was field-sown on reduced potato ridges, which were about 15 cm in height at the moment of sowing. Weed management was done using a Rumptstad speed ridger, a machine which is common practice in organic potato production to weed and earthen-up ridges in one go (Cloutier et al. 2007). For future development and fine-tuning a field-sown potato cropping system, novel weeding techniques like spot sprayers and laser-weeders—based on vision technology combined with algorithms to distinguish crop and non-crop plants (Melander et al. 2015)—could be considered for weeding, resulting in a significant decrease of inputs compared to full field applications.

Similarly, an increase in tuber yield due to increased sowing density was observed in a small test trial (19.1.4) in 2019 where TPS were sown on small ridges (half size of a conventional final ridge; see Fig. S3 for photographs over time). In these ridges, single and double rows were sown at densities of 12.1 and 24.2 seeds/m^2^, resulting in 3.0 and 5.7 *established plants*/m^2^ (*plantlet establishment ratios* of 0.244 and 0.235), respectively. To prevent tuber greening and mimic the conventional seed tuber–based potato cultivation system, ridges were earthened up during its initial growth up to the size of a conventional ridge using a Rumptstad speed ridger. On 10 October (126 days after emergence) the trial was harvested, resulting in a total yield of 17.6 and 24.0 Mg/ha for the single and double row systems, respectively. Marketable yields were 15.5 and 19.7 Mg/ha.

### Important Practices for Successful Field-sowing of Hybrid TPS Summarised

Based on these trials, seed pelleting, decent sowing-bed preparation, and irrigation are major requirements to produce a field-sown crop (Table 4). Meanwhile, special attention should go to the timing of sowing, weed management, and sowing density to increase yield.

**Table 4:** Most important factors in success of field-sowing trials from 2016 to 2019 and their effect on the critical phases of *seed germination* (**SG**), *seedling emergence* (**SE**), *plantlet establishment* (**PE**), and yield formation (**YF**). The (number of) ‘ + ’-symbols indicate how successful experiences were for a phase

Success factor	Effect in critical phase				Explanation
	**SG**	**SE**	**PE**	**YF**	
TPS pelleting	** + + + **	** + + **	** + **		o Enhancement of moisture availability o Necessary for precision sowing
Preparation of sowing bed and sowing depth	** + + **	** + + + **	** + + **		o A sowing-bed structure which ensures or improves moisture availability and temperature regulation, and which will not hamper rapid emergence o On a sandy soil, a 1–2 cm sowing depth was the equilibrium between TPS moisture-availability, temperature critical levels, and emergence-ability o Note that some soil types bring additional risks of crust formation by events of excess rain or irrigation
Additional irrigation	** + + + **	** + + + **	** + + + **	** + **	o Soil moisture availability triggers seed imbibition. This ensures root system growth and emergence of the shoot o From the plantlet establishment phase onwards, the crop becomes less sensitive to moisture stresses
Timing of field-sowing	** + **	** + + **	** + + + **	** + + **	o The most suitable timing of sowing is when field and environmental conditions allow proper growth and development of a TPS crop o The field should be well-prepared and able to carry the necessary machinery without soil compaction o Field-physical properties should be within the ranges for good growth and development o Forecasted weather should not exceed critical levels beyond which growth and development are hindered o (Too) low temperatures hinder growth, whilst (too) high temperatures (combined with wind speed, radiation and relative humidity) promote drying out of the topsoil
Weed management		** + **	** + + + **	** + + **	o Crop-weed competition should be avoided to promote undisturbed growth of field-sown TPS seedlings
Manipulation of sowing density		** + **	** + **	** + + + **	o Low plantlet establishment ratios can be tackled by increased sowing densities to increase plant density per unit area, which correlates with yield o Density manipulation allows to steer tuber yield in desired size classes, see Fig. S2

## Practical Improvements Enhancing a TPS-based Field-sowing System

To drive the advancement of the field-sowing system further and ensure productivity and yield in desirable size classes, various technological or advanced agronomical improvements can be utilised. This section provides an overview of these practical improvements.

### Seed Sorting, Priming, and Enriched Pelleting

Across trials, it was observed—although not quantified—that some of the pellets included empty, oddly shaped, or small seeds, and even non-seed materials. It is likely that these pellets would not result in successful *germination* or *seedling emergence*. Improved protocols for sorting-out abnormal seeds would have significantly contributed to the increased success in *plantlet establishment* (Benjamin 1984; Jansky and Alpers 2019).

Besides seed sorting, most seeds of field-sown cash crops undergo priming treatments to increase the seed lot’s *germination* speed, percentage, and uniformity, even under more severe field conditions (Brocklehurst et al. 1984; Devika et al. 2021). In greenhouse trials with the same experimental hybrids (van Dijk et al. 2021, 2022a, b), it was observed that *emergence* ratios were between 60 and 80%, including about 10% of the seedlings which needed more time for *emergence* (First author's personal observations, not measured or shown). Priming TPS before sowing into the field might increase seed *germination* percentage and uniformity in timing of *germination*, which will ultimately improve the uniformity of *seedling emergence*, *plantlet establishment*, and later crop stand and maturity. A priming treatment can also advance seedling vigour for rapid and healthy plantlet development (Melander and Rasmussen 2001).

The reasons for using pelleted experimental TPS in the discussed work were two-fold: (1) enable mechanical sowing with precision equipment by standardising the TPS—round—shape and increasing the size, and (2) stimulate the TPS *germination* and *emergence phase* by using clay with a high water-absorbing capacity. In multiple cash crops, seed pellets are nowadays enriched with various nutrients, macro and trace elements, to enhance *seedling emergence* and *plantlet establishment*. Even additional pellet dressings, like biostimulants (Köhl et al. 2024) are used to enhance early plant vigour and crop growth. Studying the specific properties regarding seed priming possibilities and beneficial pelleting additives for future TPS hybrid varieties might significantly increase the successes of the field-*germination*, *emergence*, and *establishment phases* of field-sown TPS.

### Enhancing a Stable Microclimate from Sowing to Plantlet Establishment

Different from a greenhouse, the conditions around a field-sown TPS are more variable. Shortage of available soil moisture between the *germination* and *plantlet establishment* *phases* is devastating for the young TPS seedlings (cf. Table 4). In Dutch agriculture, sprinklers are most frequently used to provide crops with sufficient moisture, as were also used in the described trials. However, sprinkler systems often bring cold (4–10 °C) irrigation water which reduces growth and development of tiny TPS seedlings, e.g., by temperature shocks and leaf damage.

A more technologically advanced option to manage soil moisture levels is the use of drip irrigation, which is placed 5–15 cm below soil level. A fine-mazed system releases irrigation water at low intensity towards the plant’s root system. Thus, plant and soil temperatures will be less dramatically affected compared with the large sprinkler systems, and likewise will the damage of high water intensities on young seedlings be less. Even more advanced drip systems allow for applying dissolved nutrients via the irrigation water (fertigation): a grower can precisely provide a young *emerging* and *establishing* field-sown crop with the required soil moisture and nutrients for speedy growth.

**Fig. 9:**
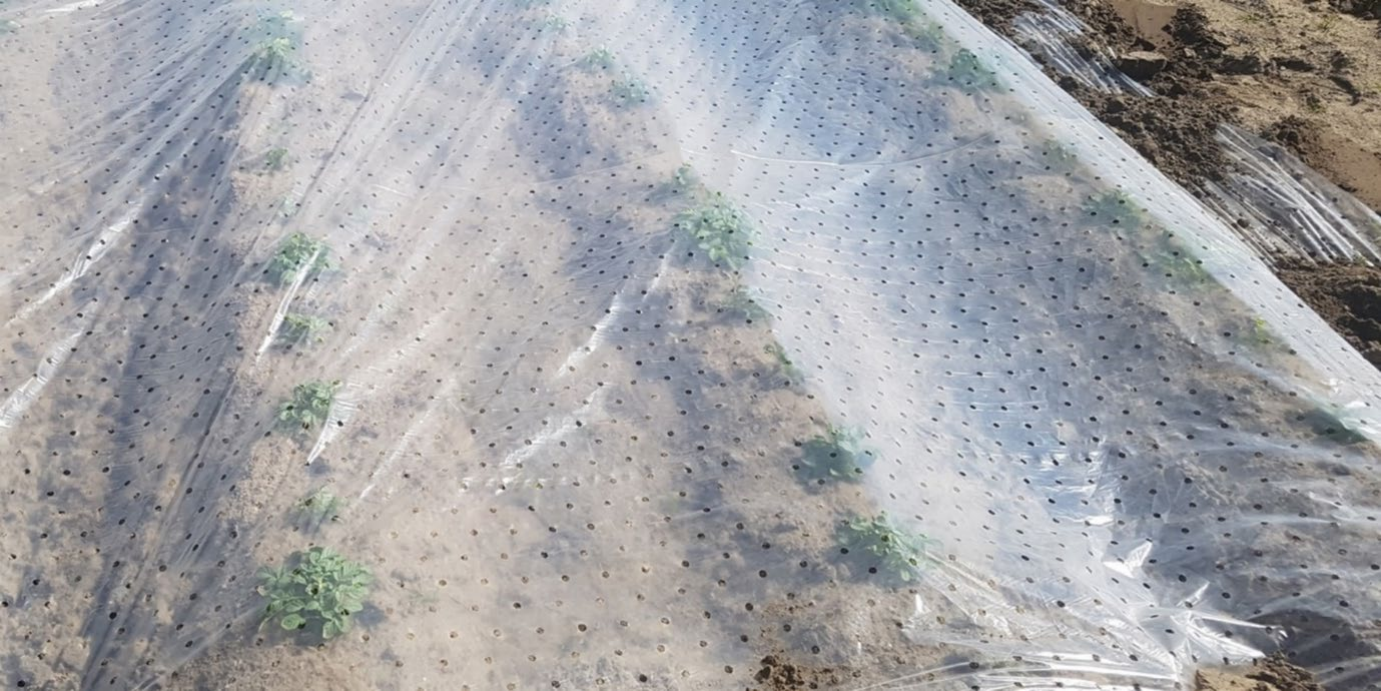
Young potato plantlets—derived from a hybrid TPS–based greenhouse nursery—covered with a plastic film to increase the temperature around the young crop to enhance initial growth and development for advancing the crop cycle

Another early-growth stimulating crop management measure is the installation of a temporary field cover (Fig. 9) by placing a plastic or fleece cover over the field after sowing—often done in vegetable production to advance the crop cycle or protect against frost—or even a plastic tunnel as applied in leek, asparagus, and strawberry field (-nurseries) (M. van Lier, personal communication, 27 March 2024).

By protecting seedlings against wind and temperature drops, but allowing radiation, a plastic cover can stabilise the microclimate around the plantlets and increase temperature around the seedlings (Villalobos et al. 2016), and thus stimulate early plant development (Bürcky 1988). The cover also prevents excessive evaporation, which results in improved soil moisture management. However, there might be negative side-effects like increased growth of weeds, increased relative humidity promoting diseases, and excessively high temperatures.

### Introducing Tailor-made Genetic Traits to Improve Early Growth and Cold Tolerance of Future Hybrid TPS

Improvement of the hybrid TPS genetics could be beneficial to build successfully a future potato cropping system, based on field-sown hybrid TPS. The genotypes sown in the discussed trials were sourced from the new diploid breeding programme of the hybrid potato start-up Solynta, without a specific breeding aim on traits beneficial for field-sowing. Since hybrid potato breeding allows for stacking multiple beneficial traits in one genotype, it should be feasible to also include specific agronomical traits, beneficial for field-sowing of hybrid TPS to ease the early seedling growth and *plantlet establishment*.

The first trait of interest should be the seed size of hybrid TPS. Large TPS will enhance early vigour as more seed reserves are available to stimulate the *seed germination* and *emergence process* (Dayal et al. 1984; Almekinders and Wiersema 1991; Almekinders 1995; Alpers and Jansky 2019). Additional seed reserves also may allow for sowing in deeper soil layers, which are less prone to drying out, as the hypocotyl could be supported for a longer period to grow towards the soil surface.

Secondly, traits regarding early root development in the *plantlet establishment phase* will be beneficial as an earlier developed and increased root system will specifically reduce the seedlings’ sensitivity to reduced soil moisture levels and will enhance nutrient availability.

The third trait of interest in novel genetics dedicated for a field-sowing system is cold tolerance (Pino et al. 2008; Fürtauer et al. 2019). Cold-tolerant hybrid potato genetics may mitigate the grower’s choice for earlier sowing, which may enhance the crop’s potential yield when having favourable growing conditions during the crop’s initial stage (Steffan and Palta 1986).

A practical example where the combination of favourable traits (e.g., early maturity) using tetraploid genotypes in a field-sown system is tested is discussed in Box 3.

**Box 3** Field-sowing for little potatoes; a practical example.

**Table:** 

The Little Potato Company was founded in 1996 in Alberta, west Canada, and started to sell home-grown little potatoes (so-called Creamers or Baby Bakers) at local farmers’ markets. Nowadays, they are a leading company in North America which breeds, grows, packages, and markets their own fresh potatoes. The company collaborates with Tuberosum Technologies Inc. which exclusively breeds new varieties for their market and short season. Their breeding programme focuses on nutrient-rich varieties which are high in vitamins and minerals and has a strong focus on consumer acceptance and experience of their potatoes as a healthy convenient food. As the company is both involved in the breeding—resulting in large amounts of TPS—and in the cultivation process—need for large numbers of small-sized potatoes—Tuberosum started pioneering in field-sowing of TPS to produce seed(ling) tubers and ultimately little potatoes for consumer markets. They believe that their focus market, small-size potatoes, is especially fit to make the first step into field-sown potato crops as small-sized potatoes can do with a shorter season and size variation is smaller compared to larger-sized potatoes grown from TPS. As their breeding programmes unlock many new varieties over time, field-sowing is a 1 to 1 replacement to transplanting greenhouse-raised plantlets for seed(ling) tuber production, and an ideal technology which is cost and labour saving, flexible and can speedily act on changing field conditions. Field-sown potato crops avoid, in their opinion, locked-up capital of seed tubers in high-technological storage houses, and decrease cultivation risks which seed tuber multipliers face every year before sufficient multiplication cycles result in a final cash-flow after selling the seed tubers. In their approach to grow seed(ling) tubers and little potatoes from a field-sown crop, the Little Potato Company and Tuberosum Technologies make use of experiences from skilled vegetable growers to modify existing vegetable machinery or develop new equipment to support the cultivation. Weed management needs more attention compared to cultivation from seed tubers; however, best practices are developed in applying right herbicide dosages and for the future the company will focus on additional mechanical weeding, including finger-weeders, combined with hilling practices. In their experience, hilling the seedlings as soon as possible without covering the seedling prevents tuber greening and stimulates stolon and tuber set, resulting in larger number of tubers per plant. Soil moisture and moderate soil temperatures are key in a decent seedling establishment and good final yield. It is claimed to keep the soil always moist up to plantlet establishment. In the future, they will focus on manipulating plant numbers by increased sowing densities to increase yields. Also, cultivation on different ridge sizes and even beds or flat grounds will be part of their applied research programme to come to a field-sown little potato for healthy consumption (J. van der Schaaf, personal communication, 26 July 2022).	

## Conclusion

From the results of multiple trials over several years, it can be concluded that field-sowing of hybrid TPS is feasible under Dutch conditions. Competitive yields of 40.6 Mg/ha were achieved in the seed tuber size classes. However, because the crops did not contain a decent share of large-sized tubers, field-sowing is not yet feasible for French fries or chips markets (Fig. S4). As novel, experimental hybrid genotypes were used in the conducted trials, it is not clear if the absence of large-sized tubers should be attributed to the genetics, the slow initial growth, or a combination of both factors. Nevertheless, a smaller-sized ware segment like Baby Bakers might be an ideal crop to produce from field-sown hybrid TPS (Box 3).

To bring field-sowing of hybrid TPS from a feasible to a practical alternative potato production strategy for the future, it is important to:

1. Decrease the risk of crop failure by increasing the *plantlet establishmen*t ratios via the means of improved agronomic practices and technologies, as well as adding tailor-made genetics in future varieties which favour a rapid initial growth.
2. Support future growers with knowledge and tools from gained practical experiences to add the useful situational awareness for cultivating a novel crop like field-sown TPS.

## Supplementary Information

Below is the link to the electronic supplementary material.

ESM 1 Two theoretical examples of temperature fluctuations in contrasting seasons during the start of the cropping cycle (Calendar week 15, 2nd week of April) under Dutch conditions. Panel **a** depicts a colder seasonal start compared to a warmer start of the season in Panel **b **(PNG 101 KB)

ESM 2 Yield produced in different tuber size classes at the final harvest in the sowing density trial (19.1.2) conducted in 2019 which was repeated twice in time, sown on 30 April and 21 May, Panel **a** and **b**, respectively. It was aimed to achieve sowing densities of 12.5, 25, 50, 100 and 200 seeds/m^2^ (D2 to D5 respectively), equal to transplant densities used by van Dijk et al. (2022a). In practice, with row-spacings of 50, 25 and 12.5 cm, five different densities of 13.8, 27.6, 47.1, 94.1 and 195.1 seeds/m^2^ were applied using the row × seeding distances 0.500 × 0.145 m, 0.250 × 0.145 m, 0.250 × 0.085 m, 0.125 × 0.085 m and 0.125 × 0.041 m respectively (PNG 47.7 KB)

ESM 3 Crop growth and development of field-sown hybrid TPS on reduced ridges, sown on 29 May 2019. One row per ridge was sown on the left four ridges and two rows per ridge were sown on the right four ridges. Pictures **a** to **d** were made on 19 July, 25 July, 29 July, and 9 September, respectively. Ridges were earthed-up to the size of a conventional ridge using a Rumptstad speed ridger (PNG 9.27 MB)

ESM 4 Harvest impression of Trials 18.1.7 (left) and 18.1.1 (right) carried out on 18 October 2018 and 16 November 2018 respectively. The left picture shows the total tuber yield of one plot (10 m^2^) including some larger sized tubers. The right picture shows the total tuber yield of one plant, sown at 18.2 seeds/m^2^ (PNG 6.14 MB)

## Data Availability

Yes.
