# Supplementary figures and images for: Is Field-sowing of Hybrid True Potato Seeds Feasible for Seed or Ware Potato Production under Dutch Conditions? An Analysis Based on a Review and Twenty Experiments
Source: Potato Res. 2025 Jul 17;68(4):3705–32. doi: 10.1007/s11540-025-09889-3 (PMC12689738; doi:10.1007/s11540-025-09889-3)

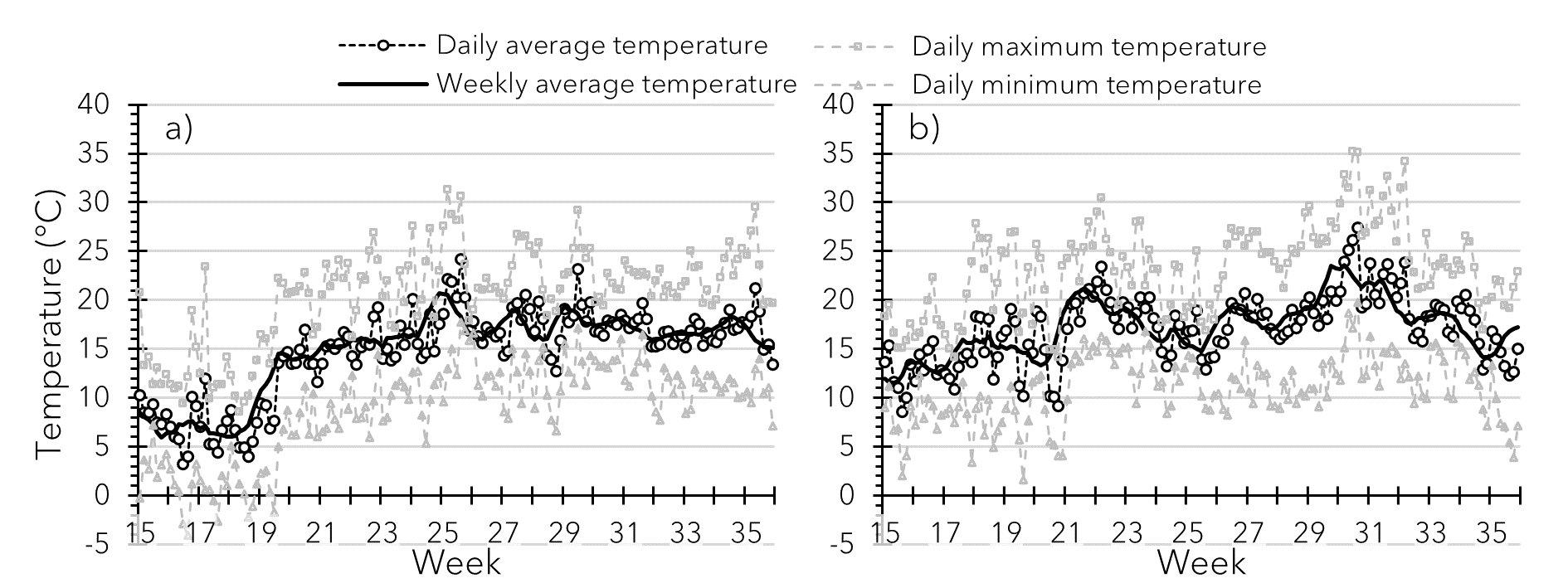

Supplement: Supplementary file 1 — Two theoretical examples of temperature fluctuations in contrasting seasons during the start of the cropping cycle (Calendar week 15, 2nd week of April) under Dutch conditions. Panel a depicts a colder seasonal start compared to a warmer start of the season in Panel b (PNG 101 KB) [file 11540_2025_9889_MOESM1_ESM.png]

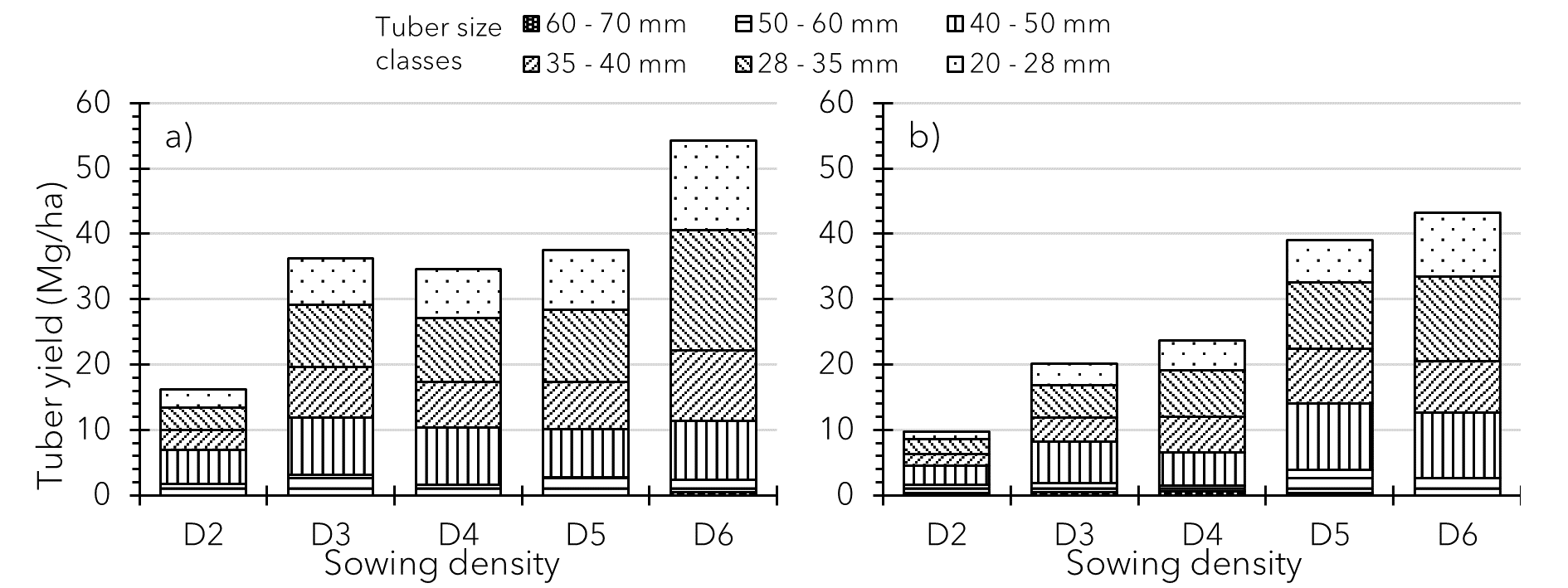

Supplement: Supplementary file 2 — Yield produced in different tuber size classes at the final harvest in the sowing density trial (19.1.2) conducted in 2019 which was repeated twice in time, sown on 30 April and 21 May, Panel a and b, respectively. It was aimed to achieve sowing densities of 12.5, 25, 50, 100 and 200 seeds/m2 (D2 to D5 respectively), equal to transplant densities used by van Dijk et al. (2022a). In practice, with row-spacings of 50, 25 and 12.5 cm, five different densities of 13.8, 27.6, 47.1, 94.1 and 195.1 seeds/m2 were applied using the row × seeding distances 0.500 × 0.145 m, 0.250 × 0.145 m, 0.250 × 0.085 m, 0.125 × 0.085 m and 0.125 × 0.041 m respectively (PNG 47.7 KB) [file 11540_2025_9889_MOESM2_ESM.png]

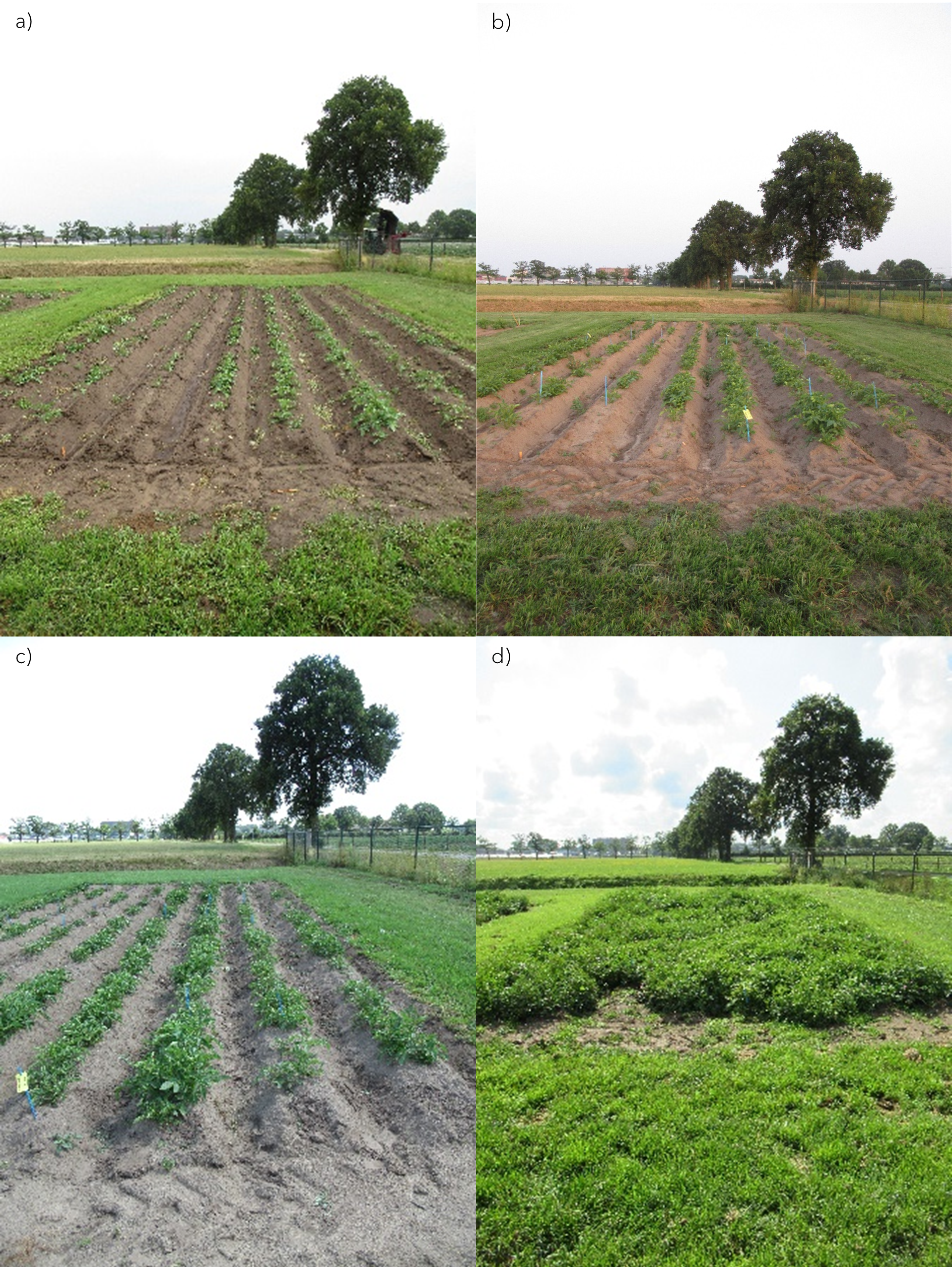

Supplement: Supplementary file 3 — Crop growth and development of field-sown hybrid TPS on reduced ridges, sown on 29 May 2019. One row per ridge was sown on the left four ridges and two rows per ridge were sown on the right four ridges. Pictures a to d were made on 19 July, 25 July, 29 July, and 9 September, respectively. Ridges were earthed-up to the size of a conventional ridge using a Rumptstad speed ridger (PNG 9.27 MB) [file 11540_2025_9889_MOESM3_ESM.png]

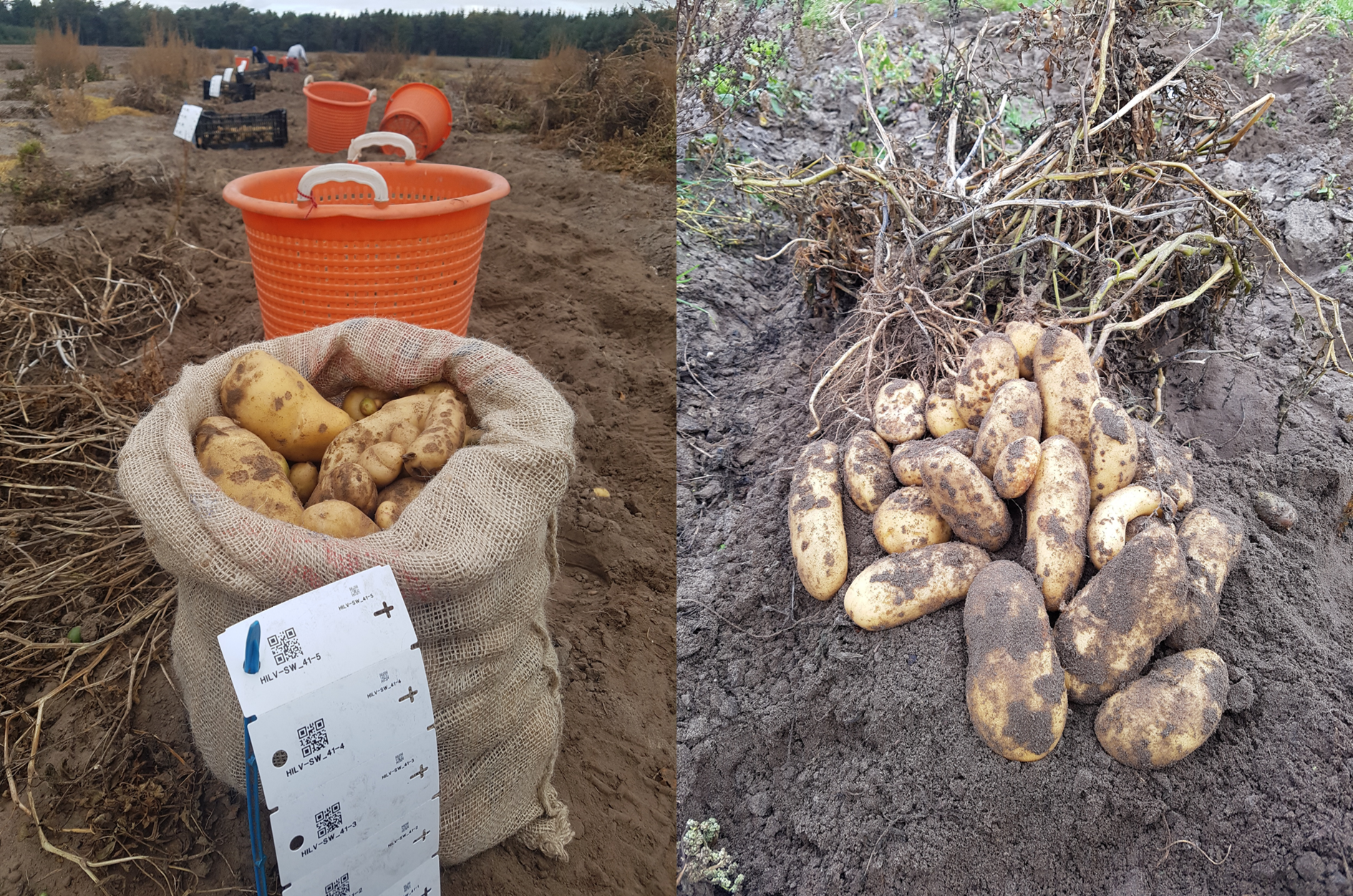

Supplement: Supplementary file 4 — Harvest impression of Trials 18.1.7 (left) and 18.1.1 (right) carried out on 18 October 2018 and 16 November 2018 respectively. The left picture shows the total tuber yield of one plot (10 m2) including some larger sized tubers. The right picture shows the total tuber yield of one plant, sown at 18.2 seeds/m2 (PNG 6.14 MB) [file 11540_2025_9889_MOESM4_ESM.png]
